# Efficient ring perception for the Chemistry Development Kit

**DOI:** 10.1186/1758-2946-6-3

**Published:** 2014-01-30

**Authors:** John W May, Christoph Steinbeck

**Affiliations:** 1 Cheminformatics and Metabolism, European Molecular Biology Laboratory-European Bioinformatics Institute (EMBL-EBI), Wellcome Trust Genome Campus, Hinxton, Cambridge, UK

**Keywords:** Rings, Cycles, CDK

## Abstract

**Background:**

The Chemistry Development Kit (CDK) is an open source Java library for manipulating and processing chemical information. A key aspect in handling chemical structures is the determination of the chemical rings. The rings of a structure are used areas including descriptors, stereochemistry, similarity, screening and atom typing. The CDK includes multiple algorithms for determining the rings of a structure on demand. Non-unique descriptions of rings were often used due to the slower performance of the unique alternatives.

**Results:**

Efficient algorithms for handling chemical ring perception have been implemented and optimised in the CDK. The algorithms provide much faster computation of new and existing types of rings. Several optimisation and implementation considerations are discussed which improve real case usage. The performance is measured on several publicly available data sets and in several cases the new implementations were found to be more than an order of magnitude faster.

**Conclusions:**

Algorithmic improvements allow handling of much larger datasets in reasonable time. Faster computation allows more appropriate rings to be utilised in procedures such as aromaticity. Several areas that require ring perception have also seen a noticeable improvement. The time taken to compute the unique rings is now comparable allowing a correct usage throughout the toolkit. All source code is open source and freely available.

## Background

The Chemistry Development Kit (CDK) [1,2] is an open source Java library for manipulating chemical information. A key aspect of manipulating and querying chemical information is the ability to define and reason about attributes of chemical structures. Describing the rings in a structure is fundamental and a prerequisite of other attributes.

There is often a disconnect between how chemical rings are numbered and what is useful for computation. Conflicting definitions of rings contribute towards discrepancies between chemistry toolkits such as assigning aromaticity. The CDK does not provide a single strict definition of what rings are present in a structure. The ring information is considered auxiliary with different algorithms utilised for a specific use-case. Some considerations of the differences will be touched upon but a thorough review is provided by [3,4] and [5].

There are several key properties we wish to know: is an atom or bond in a ring, what size is the ring and what are the other atoms and bonds in the ring? This information can be stored as an attribute of each atom or bond, as a collection of rings on the structure or computed on demand. With the provision of multiple algorithms it is undesirable to store all the information but invariant properties including membership and smallest ring size could be stored as an attribute of an atom or bond.

The ring properties can be used in many procedures throughout the library. In similarity searching and screening the creation of chemical fingerprints [6] may include ring size or membership to reduce the number of false positives. When matching atoms and bonds between structures the ring properties can be used in early elimination of infeasible matches or to disfavour ring opening and closing. Ring properties are also utilised in structure patterns (SMARTS [7]) where ring membership, size and number of rings can be queried.

It is essential that different structure resonance forms are treated as equivalent, one approach is to treat bonds in aromatic ring systems as delocalised. Conversely a delocalised structure may have been provided without specified bond orders. The ring properties can be used to localise and delocalise the bonds between aromatic and Kekulé representations.

Geometric isomers (double-bond stereochemistry) should not be encoded when the bond is involved in a rigid ring. Rigidity is approximated by only allowing stereoconfigurations in rings with more than seven atoms. Groups of interdependent stereocenters can be identified by recursively checking the rings in a structure [8].

Improving the core ring perception algorithms can influence many areas and it is important that efficient algorithms are used.

### Graph theory preliminaries

Although more comprehensive and accurate methods exist, chemical structures can be represented and efficiently modelled as graphs [9]. The algorithms used for ring perception are not specific to chemical structures and require several formal definitions. The basic concepts for these are briefly introduced here. A graph is composed of a set of vertices *V* and a set of edges *E*. Each vertex or edge may be *labelled* with a value. Two vertices are *adjacent* if an edge exists which contains the two vertices. The vertices of an edge are known as the endpoints, each endpoint is said to be *incident* to the edge. A *degree* of a vertex is the number of incident edges. If the endpoints are unordered, an edge is said to be *undirected*. Simple graphs have no edges connecting the same vertex (loops) and no edges which share the same endpoints (multiedges). We model a chemical structure as simple undirected labelled graph where the atoms and bonds are labels on the vertices and edges. Although the edges have a numeric value (bond order) they are not treated as weighted.

A *walk* is a sequence of vertices and edges connecting two vertices. If the start and end of the walk are the same, the walk is *closed*. Otherwise the walk is *open*. A *walk* is *simple* if it contains no repeated edges and *elementary* if there are no repeated vertices. A *simple* walk that is also *open* it is referred to as a *path*. Two vertices are *connected* if there is a *path* between them. A graph is *connected* if each vertex can be reached from every other vertex. A *connectedcomponent (ConnComp(G))* in an undirected graph is a subgraph in which every vertex is *connected*.

A *cycle* is a closed *walk*. Graphs containing a *cycle* are said to be *cyclic* or *acyclic* if no cycle is present. Acyclic simple graphs are referred to as a *tree*. A ring in a chemical structure is best described as an *elementary* cycle. The cycle has no repeating vertices or edges and each vertex has a *degree* of 2 (in the cycle). This definition includes envelope rings of structures like napthalene and azulene. As we are primarily concerned with chemical structures herein we use the term *cycle* to refer to *elementarycycle*.

A *cyclebasis* is a set of cycles which can be used to generate all other cycles (cycle space) of the graph. Representing a cycle as a set of edges, a new cycle can be generated using the symmetric difference (XOR, ⊕-summing) of the edge sets of two cycles whose edge sets intersect. A *minimumcyclebasis* is a cycle basis of minimum weight, in an unweighted graph the weight is simply the number of edges. When there is more than one basis with the same weight the choice between them is arbitrary as either can be used to generate the cycle space.

### Cycle membership

The first step in cycle processing for a chemical structure is to efficiently determine which vertices and edges of the graph belong to a cycle. In PubChem-Compound [10] (Aug 2013) 97.3% of structures (47,745,887) contained a cycle. Although the proportion of structures containing a cycle is high only 59.3% of the heavy atoms and 57.3% of bonds were cyclic. Eliminating these acyclic vertices and edges from further processing reduces the size of the computation.

The SpanningTree was introduced in the CDK to eliminate acyclic vertices and edges, reducing the runtime of existing algorithms [11]. A graph *H* is a subgraph of a graph *G* if the vertices *V* and edges *E* of *H* are a subset of *G*. A subgraph *G* is said to be a *spanningsubgraph* of *H* if every vertex of *H* is present in *G*. The edges in chemical structures are unweighted and so the minimum spanning tree is a tree with the smallest number of edges. Given an input structure a spanning tree is created which contains a subset of the edges that span the vertices but contains no cycles. The SpanningTree class uses a greedy algorithm [12] to sequentially build up this tree. Cyclic vertices and edges are determined by finding a path in the tree between the two endpoints of an edge which was not included. Any edge that is not in the spanning tree is cyclic and any path in the tree which connects the two endpoints contains vertices and edges that are also cyclic. The number of paths to find depends on the number of edges not included in the spanning tree. Structures containing a large number of rings will have more edges removed and more paths to find. Discovery of a path in the tree is implemented as depth-first-search and the entire tree may be traversed for each removed edge.

### Cycle sets

In addition to determining if a vertex or edge is cyclic, one would also like to know the sizes of cycles and the *walks*. There is an exponential number of elementary cycles in a graph and smaller subsets of this have subsequently been defined and used in various aspects of chemical information processing.

### Smallest set of smallest rings/minimum cycle basis

**Figure 1:**
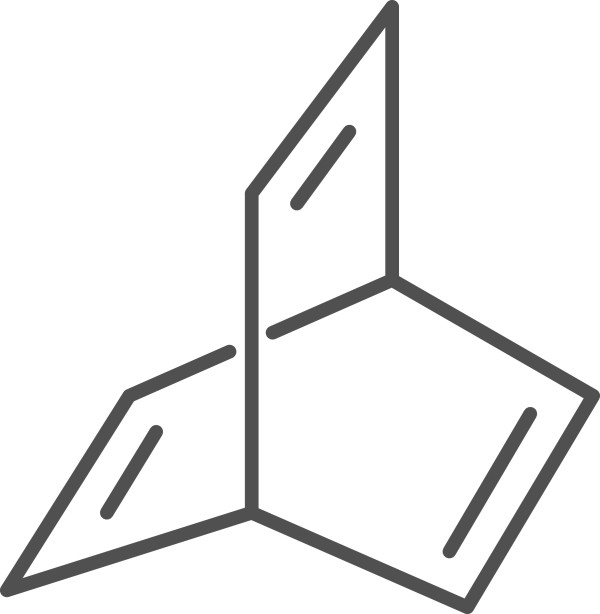
**The structure of barrelene.** Barrelene is thought of having two rings and mirrors what is found in the MCB and reflected in its systematic name (bicyclo[2.2.2]octa-2,5,7-triene). The choice of which two rings is arbitrary. Barrelene has three relevant cycles and no essential cycles. In this simple example the choice of which two cycles is irrelevant as they are symmetric. This would no longer be the case if the structure was hetereocyclic or had exocyclic group added.

A well known set of cycles is the Smallest Set of Smallest Rings (SSSR). The SSSR was originally defined as a minimum length Kirchhoff-fundamental basis but has evolved to refer to a minimum cycle basis (MCB). The original definition of SSSR does not always contain the shortest cycles and was computationally intractable [3]. To avoid confusion the term SSSR will now only be used in reference to CDK implementation names. As introduced previously the MCB is a polynomial set of cycles which can be used to generate the cycle space. As the MCB may not be unique it has little direct use in similarity, aromaticity, depiction or other descriptive features. It is also not required to find the shortest cycle through each edge or vertex which can be accomplished without checking the cycles form a basis. Although the MCB is not unique, the number of cycles it contains is. This value is the circuit rank^a^ and is the number of edges that would need to be removed to make the graph acyclic (a spanning tree). For these reasons the size of the MCB agrees with de-facto standards and chemical nomenclature (Figure 1). The formula |*E*|−|*V*|+|*ConnComp*(*G*)| provides the circuit rank without computation of the cycle walks [5].

The original algorithm [13] utilised in the CDK was shown to be incorrect and can not guarantee completion on all graphs [3]. Although one may consider such cases rare in four of the five tested chemical data sets (Table 1) at least one structure was found which caused the CDK implementation to halt indefinitely. The algorithm is still partially used in other cheminformatics libraries [14]. The implementation was replaced with a correct algorithm [3] (SSSRFinder) which also provides uniquely defined cycle sets as alternatives to the MCB.

**Table 1:** Chemical structure sets used to measure performance

**Identifier**	***n*** **structures**	**Description**	**Available**
chebi_108	26,790	ChEBI Release 108 [15]	http://www.ebi.ac.uk/chebi
nci_aug00	250,172	NCI Aug 2000 [16]	http://cactus.nci.nih.gov/download/nci
zinc_frag	504,074	Zinc Clean Fragments	http://zinc.docking.org/subsets/clean-fragments
		Ph7 2013-04-12 [17]	
chembl_17	1,318,180	ChEMBL Release 17 [18]	http://www.ebi.ac.uk/chembl
zinc_leads	5,135,179	Zinc Clean Leads Ph7 2013-05-31 [17]	http://zinc.docking.org/subsets/clean-leads

The number of structures is the number which were successfully read from SMILES [19] notation. The ChEBI and ChEMBL datasets had a small number erroneous SMILES string which could not be interpreted.

In general the CDK library has been relying less on MCB as it has little use beyond counting the number of rings and generating the cycle space. Both of these tasks can be achieved more efficiently with other procedures. The implementations provided in the CDK are primarily for reference and their use in computing other uniquely defined cycle sets.

### Essential and relevant cycles

The essential and relevant cycles are a uniquely defined set of cycles. The MCB is non-unique when there are multiple minimum cycle bases and an arbitrary choice of a single basis can generate the cycle space. The essential cycles is the intersect of these minimum cycle bases whilst the relevant cycles is the union. When a graph has a single unique MCB it is equal to both the essential and relevant cycles. As a subset of the MCB the essential cycles do not form a basis and cannot be used to generate the cycle space. Like the MCB the essential cycles are always polynomial in number. Counter-intuitively, structures such as barrelene (Figure 1) contain no essential cycles. The relevant cycles do form a basis but may be exponential in number.

The uniqueness of these cycle sets make them desirable for describing chemical entities. The essential cycles have been utilised in the CDK for similarity searching techniques including generation of fingerprints and for the structure query patterns. Unfortunately the computation of the unique essential and relevant cycles (using the SSSRFinder) takes much longer than the non-unique MCB. The increased computation runtime has generally meant the MCB has been favoured.

### All elementary cycles

**Figure 2:**
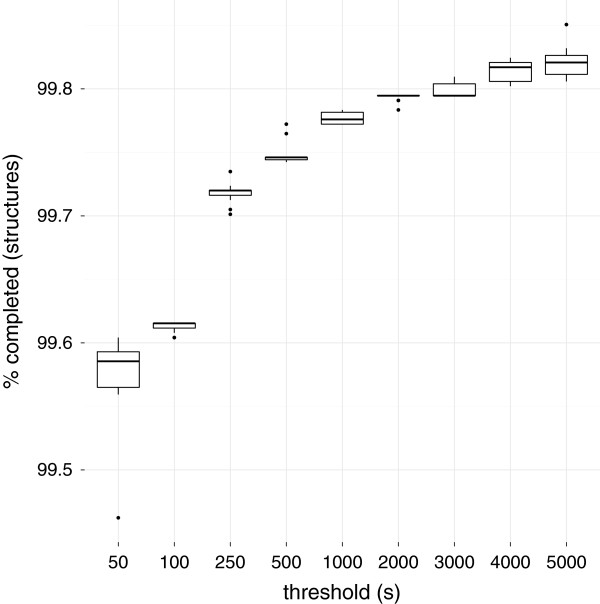
**Coverage of different timeout thresholds when finding all elementary cycles.** The percentage of structures in ChEBI 108 [15] that the AllRingsFinder successfully finished was measured for different timeout thresholds. Increasing the threshold larger to more than a second has minimal impact.

**Figure 3:**
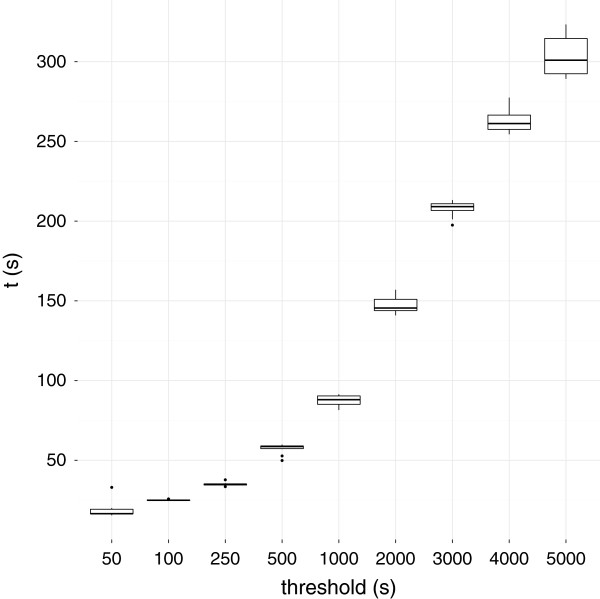
**Time taken of different timeout thresholds when finding all elementary cycles.** The time taken for ChEBI 108 [15] to be processed by the AllRingsFinder was measured for different timeout thresholds.

When considering all cycles, the number of cycles can be very large and infeasible to compute for fullerene-like and cyclophane-like structures. The set of all cycles can be generated using a cycle basis or computed directly [20]. Direct computation is more efficient and is provided in the CDK as the AllRingsFinder. One major drawback of the existing implementation is the dependence on a time measure to determine feasibility. The time was measured from when the algorithm started and aborted if the elapsed time exceeded a set threshold. Whether the algorithm completes then depends on the machine specification and also the current load on the processor. The *timeout* was also generally left at a value too high (5 seconds) for larger datasets. To demonstrate this, the timeout threshold was varied and tested on a small dataset. The number of structures that the algorithm successfully completed was measured. Increasing the threshold to longer than a second provides only a small gain in coverage (Figure 2). A timeout of just 50 ms allowed 99.4% of the structures to complete in 32 seconds. Leaving the timeout at the default value of 5000 ms allowed 99.8% of structures to complete but took nearly 10 times longer (291 seconds) (Figure 3). This could be an artefact of hardware improvements but highlights the difficulties in choosing an appropriate value when using a timeout. The set of all cycles was used throughout the library in fingerprint generation, similarity searching [21], descriptors, kekulisation and fragmentation. The cycles were also partially utilised in aromaticity perception.

## Implementations

**Figure 4:**
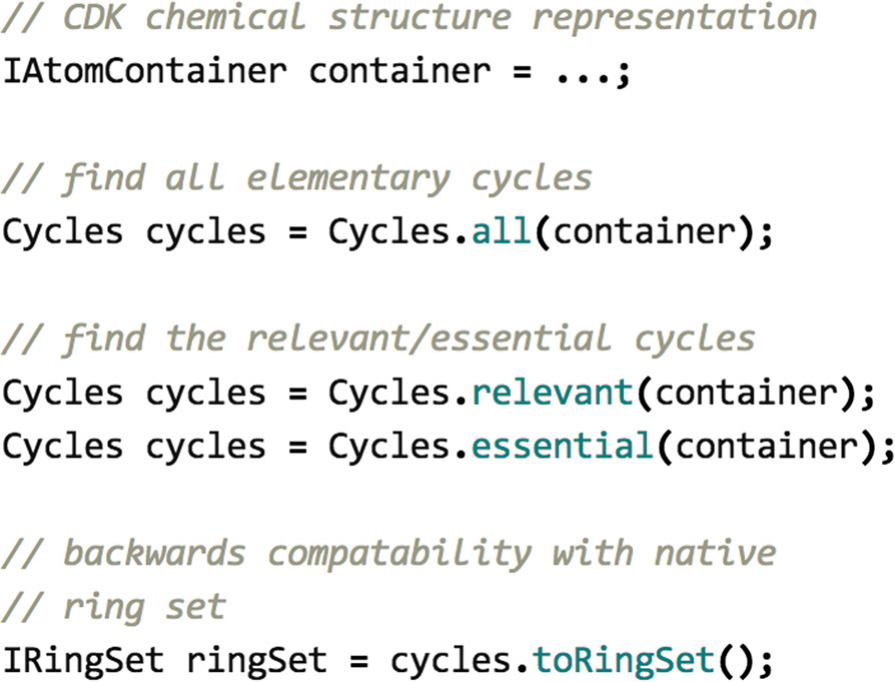
**Cycles API.** Each algorithm is split into separate classes allowing an expert users to assemble required cycle sets. The front-end Cycles facade provides simplified interaction applying optimisations as required.

The processing of cycles in the CDK has been streamlined and optimised. Improved algorithms for determining cycle membership and the uniquely defined essential and relevant cycles have been implemented in the CDK library. The algorithms are split across several classes allowing an expert user to pick and choose. For simplicity a facade, Cycles (Figure 4), provides generation of the cycle sets described and applies preprocessing optimisations. Specific implementation details are discussed and measured in the results.

### Graph representations

**Figure 5:**
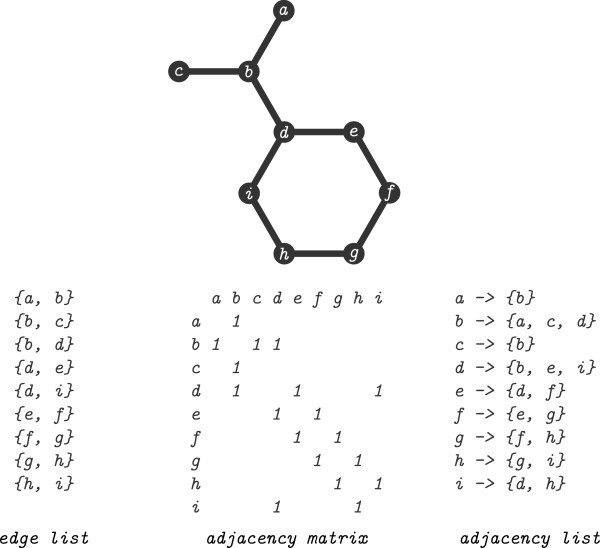
**Graph representations.** An abstract graph representation of a chemical structure (propan-2-ylbenzene) and different representations. The labelled vertices (*a*,*b*,…*i*) correspond to the values in the representations.

A graph can be represented and stored using several data structures [22] (Figure 5). The choice of data structure can dramatically affect performance. A coordinate or edge-list representation stores the vertices and edges as separate lists. The edge list is memory efficient but inefficient to determining adjacency where every edge must be checked. An adjacency matrix is a square matrix with a boolean value indicating whether two vertices are adjacent. Matrix representations offer constant time adjacency checking but require every vertex to be checked in order to obtain a list of neighbours or degree. The matrix representation is less memory efficient and requires quadratic space to store. In an adjacency/incidence list each vertex stores adjacent vertices or incident edges. Testing adjacency is bounded by the number of adjacent vertices, the *degree*[22]. The *degree* and the set of adjacent vertices can be obtained in constant time.

The choice of data structure depends on properties being modelled, and which algorithms will be used. Chemical structures are generally small (|*V*|<100) and each vertex is only adjacent to a few other vertices (sparse). Although more costly in memory and for modifications the attributes of chemical structures make the adjacency (or incidence) list representation preferable.

The CDK currently uses an edge list representation to store chemical structures. Conversion of the CDK native data type to an adjacency list is relatively quick but can become significant if carried out multiple times. Many of the existing algorithms used an optimised representation but benefit was seen by avoiding the slower CDK native objects and minimising reconversion. The overhead introduced for converting the CDK objects (Table 2) could be minimised by loading directly into an more optimal data structure. When comparing to existing methods the conversion time is included in comparisons.

**Table 2:** **Average (*n* = 15) time taken to convert CDK structure representations to adjacency and incidence list data structures**

**Chemical structure**	***n* structures**	**Adjacency list**		**Incidence list**	
		*t* (ms)	sdev	*t* (ms)	sdev
chebi_108	26,790	167	14	238	15
nci_aug00	250,172	998	49	1,347	53
zinc_frag	504,074	1,466	13	2,029	29
chembl_17	1,318,180	8,308	33	11,977	246
zinc_leads	5,135,179	22,537	582	33,567	2368

## Results and discussion

Here we describe the optimisations and measure the performance on several chemical datasets (Table 1). All measurements were performed on a 2.66 GHz Intel Core i7 processor using Java version 1.7.0_21. The unprocessed benchmark results are provided as Additional files 1, 2 and 3.

### Cycle membership

The existing algorithm used in SpanningTree was for graphs with weighted edges [12]. In an unweighted graph any spanning tree is the minimum spanning tree. A spanning tree in an undirected, unweighted graph can be constructed with a depth- or breath-first-search [23]. Although efficient in construction, the spanning tree still requires additional operations to determined the cyclic vertices and edge. A more efficient approach is to compute the biconnected (2-connected) components of the graph. The biconnected components can be found using a single depth-first search [24]. A vertex is biconnected if removing it from the graph does not increase the number components. A *biconnectedcomponent* is a maximal connected subgraph where every vertex is biconnected. In addition to detecting the cyclic vertices and edges the procedure also partitions the graph in to separate components which correspond to a separate ring systems in the chemical structure. If the number of edges is equal to the number of vertices, |*E*|=|*V*|, then the circuit rank is 1 and the component is an elementary cycle. Such components correspond to the isolated and spiro ring systems in a chemical structure whilst the other biconnected components are the fused and bridged ring systems. The simple elementary cycles need no further processing and can be skipped from the more computationally intensive algorithms.

**Figure 6:**
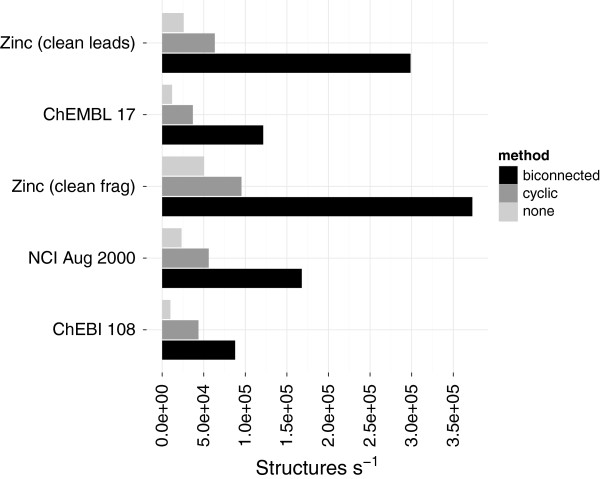
**Preprocessing cycles.** The impact of varied preprocessing when computing the minimum cycle basis (MCB). *None* indicates the MCB was computed on the entire graph whilst *cyclic* indicates the MCB was computed on a subgraph of only the cyclic vertices and edges. The *biconnected* preprocessing computed the MCB only on the biconnected component which were not simple elementary cycles. The performance includes the both the application of the preprocessing (i.e. finding the biconnected components) and the computation of the MCB. The time taken to convert the CDK objects (Table 2) is not included.

To measure the impact by utilising the biconnected components the time taken to compute the MCB was measured on the chemical structure sets. Although processing only the cyclic vertices and edges improves performance an even larger gain can be seen by processing only the non-simple biconnected separately (Figure 6). The largest performance improvement was seen for the zinc data sets that contain fewer fused ring systems.

The biconnected components were already used internally for other cycle computations (SSSRFinder). A new RingSearch utility was written with an algorithm optimised for small graphs using binary sets. The implementation provides logical testing of cycle membership for vertices and edges as well as partitioning the components and creating fragments of the input structure.

**Figure 7:**
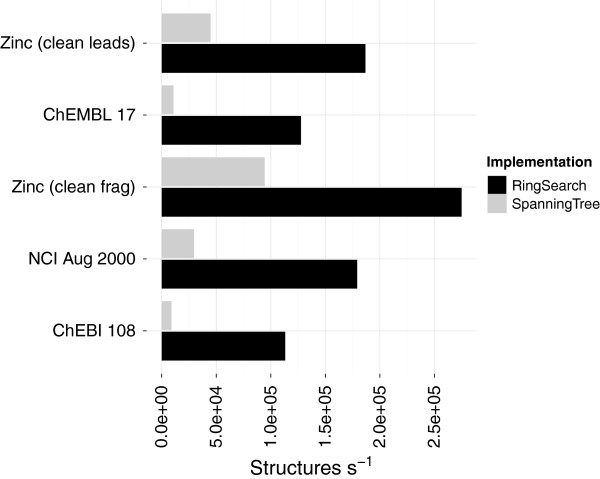
**Improved cycle membership perception.** The performance (structures per second) when using the RingSearch compared to the pre-existing SpanningTree. Times measured for the RingSearch include the conversion from the CDK objects to an *adjacency* list representation (Table 2).

The time taken on several chemical datasets showed the new implementation performs well compared to the SpanningTree (Figure 7). The new algorithm can process between 100,000 and 300,000 structures per second where the majority of time (Table 3) was spent in the conversion (Table 2). The zinc_leads data has almost five times the number of structures than chembl_17 but the SpanningTree actually finished in less time (Table 3). This is because chembl_17 contains more fused-ring systems that cause a bottle neck in processing. The difference is emphasised by measuring the performance on single structures containing large fused ring systems (Table 4).

**Table 3:** **Average and median (*n* = 15) time taken to determine ring membership**

**Chemical structure**	**SpanningTree**			**RingSearch**		
	**mean**	**median**	**sdev**	**mean**	**median**	**sdev**
	***t* (ms)**	***t* (ms)**		***t* (ms)**	***t* (ms)**	
chebi_108	2,969	2,826	246	236	212	90
nci_aug00	8,440	8,451	59	1,396	1,372	88
zinc_frag	5,338	5,353	77	1,833	1,818	60
chembl_17	122,357	122,493	616	10,325	10,303	98
zinc_leads	114,710	115,067	837	27,496	27,502	215

Times exclude IO but include conversion. It should be noted that the majority of the time spent in RingSearch was for the conversion (*adjacency*). Unprocessed measurements are provided in Additional file 1: Cycle membership benchmark.

**Table 4:** **Average (*n* = 50) time taken to determine ring membership in several complex structures from ChEBI and FULLERENE [**[25]**]**

**Chemical structure**	**SpanningTree**		**RingSearch**	
	***t* (ms)**	**sdev**	***t* (ms)**	**sdev**
cubane (CHEBI:33014)	1.18	1.24	0.21	0.66
dodecaboride (CHEBI:51706)	1.11	0.80	0.05	0.01
octacontaboron (CHEBI:50252)	100.18	52.87	0.44	0.18
C _60_ fullerene (CHEBI:33128)	11.15	2.92	0.30	0.03
C _70_ fullerene (CHEBI:33195)	10.44	4.25	0.88	0.23
C _320_ fullerene (FULLERENE)	423.06	166.30	0.61	0.32
C _720_ fullerene (FULLERENE)	3100.71	1171.62	1.65	0.66

### Minimum cycle basis

**Figure 8:**
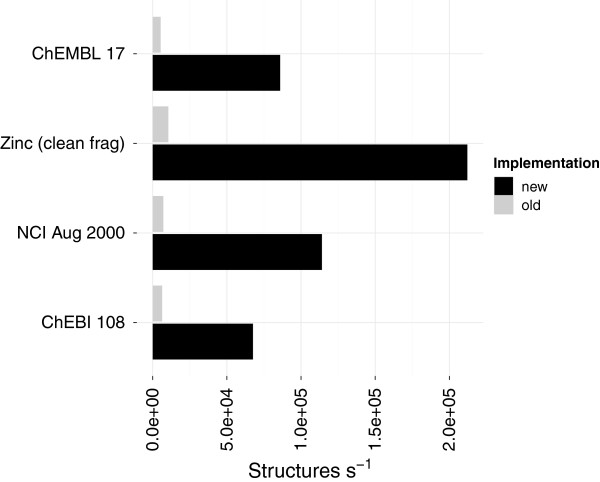
**Performance of computing minimum cycle basis.** A comparison of computing the Minimum Cycle Basis (MCB) using the old implementation (SSSRFinder) compared to the new implementation. The performance of the old implementation on zinc_leads data set was not stable. The performance includes conversion to an *adjacency* list representation for the new method (Table 2).

The new implementation MinimumCycleBasis computes the MCB as by-product of the relevant cycles [26]. Although this algorithm has a higher computational complexity than the existing (SSSRFinder) in practice it was found to be several factors faster (Figure 8). Although the existing implementation scales better, processing small graphs and an optimised implementation with fewer overheads can run faster.

The absolute time taken for processing structures from zinc_frag has dropped from ∼47 to ∼2 seconds whilst processing chembl_17 went from ∼4 minutes to ∼15 seconds (Table 5). The algorithm to compute the MCB and relevant cycles [26] first computes an initial set of cycles in a compact representation that is then reduced to either a MCB or the relevant cycles. Determining the initial set of cycles uses a shortest paths procedure where an ordering is imposed on vertices [27]. With the vertices ordered by degree if the biconnected component is known to be a non-simple component (i.e. fused or bridged) then only shortest path searches from vertices with deg >2 will yield new cycles to add to the initial cycles. In practice this reduces the number of shortest path searches, for example in the common naphthalene and anthracene substructures from 10 and 14 to 2 and 4 respectively.

**Table 5:** **Average and median (*n* = 15) time taken to compute the minimum cycle basis (MCB) using the existing and improved implementations**

**Chemical structure**	**MCB (old)**			**MCB (new)**		
	**mean**	**median**	**sdev**	**mean**	**median**	**sdev**
	***t* (ms)**	***t* (ms)**		***t* (ms)**	***t* (ms)**	
chebi_108	4,200	4,020	695	396	353	160
nci_aug00	34,762	34,330	1,685	2,193	2,125	211
zinc_frag	47,752	47,844	1,982	2,376	2,330	153
chembl_17	245,620	245,592	990	15,341	15,257	311
zinc_leads	-	-	-	-	-	-

The time taken by the new implementation includes *adjacency* conversion (Table 2). Unprocessed measurements are provided in Additional file 2: Short cycles.

The cycle basis is formed by incrementally adding candidate cycles of increasing size. In this case the candidates are the initial set of cycles [26]. A candidate is added to the basis if it is linearly independent from the current members of basis [27]. This check for linear independence is expensive^b^ and can be avoided under some conditions. With the union of all edges in the basis (*E*_*B*_) a new cycle is linearly independent if any edges of the candidate (*E*_*cand*_) are not present in basis. That is, when |*E*_*B*_∩*E*_*cand*_|<|*E*_*cand*_|, the cycle must independent. Additionally we know the basis is complete when the number of cycles is equal to the circuit rank. As the biconnected components are processed separately, the circuit rank of the component is |*E*|−|*V*|+1.

### Essential and relevant cycles

**Figure 9:**
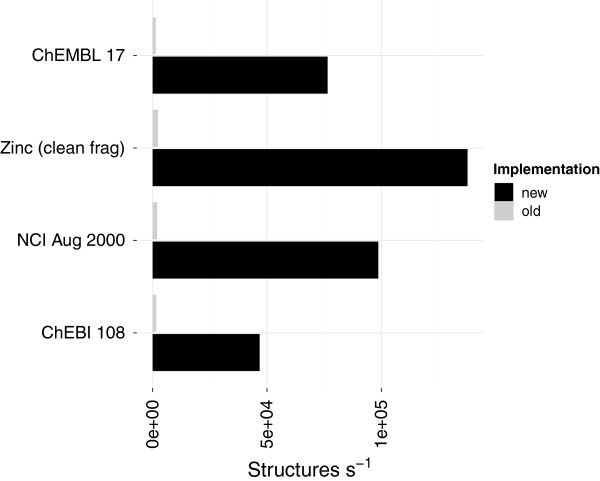
**Performance of computing relevant cycle basis.** A comparison of computing the relevant cycle basis using the old implementation (SSSRFinder) compared to the new implementation. The performance of the old implementation on zinc_leads data set was not stable. The performance includes conversion to an *adjacency* list representation for the new method (Table 2).

**Figure 10:**
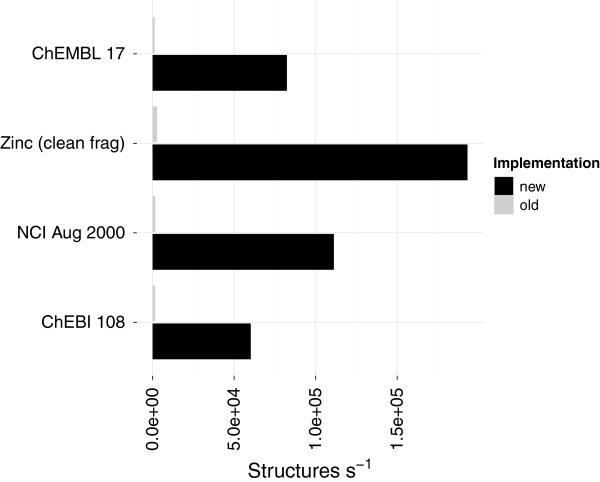
**Performance of computing essential cycles.** A comparison of computing the essential cycles using the old implementation (SSSRFinder) compared to the new implementation. The performance of the old implementation on zinc_leads data set was not stable. The performance includes conversion to an *adjacency* list representation for the new method (Table 2).

The new implementation computes the relevant cycles directly [26]. The MCB and essential cycles are then derived as a by-product. The computation of relevant and essential cycles showed a larger improvement over the existing implementations than for the MCB computation (Figures 9, 10). The old implementation could process around 2,000 structures per second whilst the newer implementations can process between 50,000 and 200,000. The time taken to find all the relevant cycles in chembl_17 has dropped from ∼16 minutes to only ∼16 seconds (including 8 seconds spent in conversion) (Table 6). Similarly the time taken to find the essential cycles in the nci_aug00 dataset went from ∼2.5 minutes to ∼2 seconds (Table 7).

**Table 6:** **Average and median (*n* = 15) time taken to compute the relevant cycles using the existing and improved implementations**

**Chemical structure**	**Relevant cycles (old)**			**Relevant cycles (new)**		
	**mean**	**median**	**sdev**	**mean**	**median**	**sdev**
	***t* (ms)**	***t* (ms)**		***t* (ms)**	***t* (ms)**	
chebi_108	17,013	16,897	576	445	388	171
nci_aug00	149,210	148,231	12,190	2,250	2,187	195
zinc_frag	183,587	184,720	18,219	2,610	2,519	237
chembl_17	972,493	972,605	1,197	16,003	15,991	201
zinc_leads	-	-	-	-	-	-

The time taken by the new implementation includes *adjacency* conversion (Table 2). Unprocessed measurements are provided in Additional file 2: Short cycles.

**Table 7:** **Average and median (*n=15*) time taken to compute the essential cycles using the existing and improved implementations**

**Chemical structure**	**Essential cycles (old)**			**Essential cycles (new)**		
	**mean**	**median**	**sdev**	**mean**	**median**	**sdev**
	***t* (ms)**	***t* (ms)**		***t* (ms)**	***t* (ms)**	
chebi_108	16,561	16,395	615	572	424	451
nci_aug00	128,963	128,663	2,325	2,536	2,459	362
zinc_frag	217,312	217,016	940	3,662	3,574	336
chembl_17	954,954	952,437	20,698	17,235	17,171	293
zinc_leads	-	-	-	-	-	-

The time taken by the new implementation includes *adjacency* conversion (Table 2). Unprocessed measurements are provided in Additional file 2: Short cycles.

**Figure 11:**
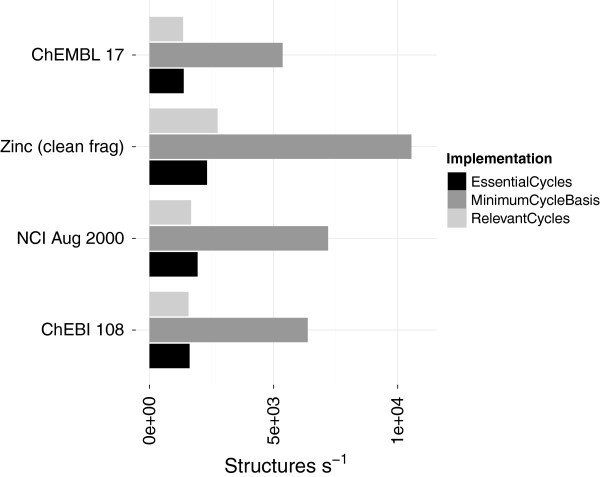
**Performance comparison of the old MCB, Essential and Relevant cycles.** Previously the computation of the MCB is much faster than the essential and relevant cycles. This led to the MCB being favoured for use in other procedures.

**Figure 12:**
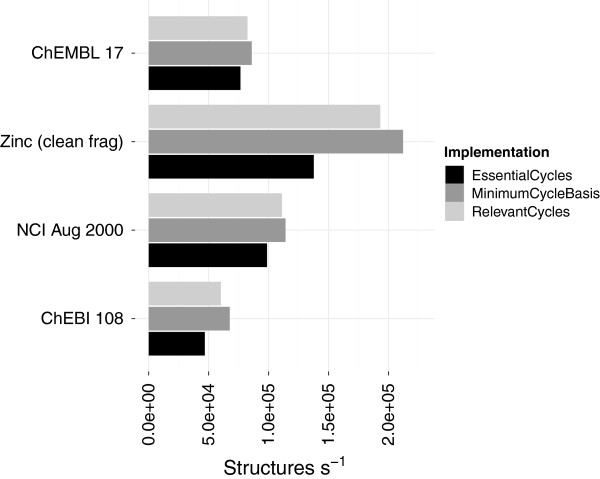
**Performance comparison of the new MCB, Essential and Relevant cycles.** The computation of the unique sets is now comparable to the minimum cycle basis. The performance includes conversion to an *adjacency* list representation (Table 2).

Previously the time taken to find the uniquely defined cycle sets was much longer than the non-unique MCB (Figure 11). The new implementation now finds the unique cycle sets in time comparable to the non-unique MCB (Figure 12).

The number of cycles found by each set was also measured on the data sets (Table 8). On average the number of cycles found in the unique sets was within 1% of the non-unique MCB. This means in practice the unique sets can readily be used as a replacement for the non-unique MCB. It is however inevitable that some structures will have an infeasible number of relevant cycles. During testing, a structure in PubChem-Compound (CID 53389303) was found to contain over 1 million relevant cycles. As the number of relevant cycles can be determined without generating the walks, such structures can be filtered out if desired.

**Table 8:** The number of cycles in each set

**Chemical**	***n* structures**	**MCB**	**Essential**	**Relevant**	**All**
**structure**					
chebi_108	26,790	56,572	55,687	57,401	∼126,713
nci_aug00	250,172	599,876	591,144	606,045	∼1,007,643
zinc_frag	504,074	880,296	875,801	882,393	∼1,022,498
chembl_17	1,318,180	4,505,285	4,455,907	4,563,027	∼6,599,942
zinc_leads	5,135,179	-	-	-	∼14,816,752

The counts for all elementary cycles are approximate as it was infeasible to finish computation on some structures.

### All elementary cycles

The data structures of AllRingsFinder were optimised and the timeout replaced with a threshold specific to the algorithm [20]. The improvements to the data structures involved representing the path-graph as an incidence-list and using binary sets to test intersection. The algorithm progresses by iteratively reducing (removing) vertices – the order of removal can be predetermined or dynamic. Using a predetermined order the edges need only be indexed by the next endpoint (i.e. directed). This reduces the number of modifications to the path-graph. Edges are only removed when a vertex is being reduced and all edges can be removed at once from this vertex. As each vertex is reduced the degree on the adjacent vertices may increased. Limiting the maximum degree the algorithm is allowed to reach provides a better threshold to determine feasibility.

**Figure 13:**
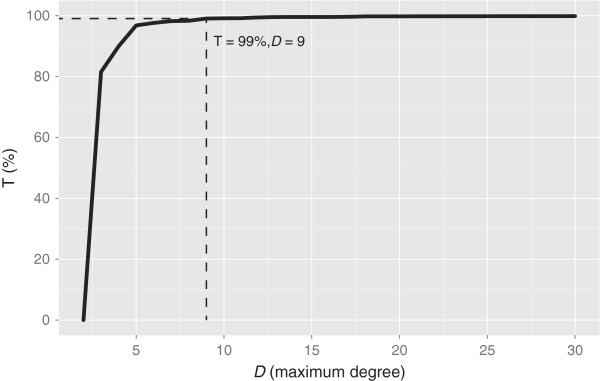
**Degree threshold required to perceive percentage of fused ring systems.** Percentage (*T*) of feasible ring systems in PubChem Compound (Dec 2012) for a given degree (*D*).

To determine an appropriate threshold the algorithm was run on all fused ring systems found in PubChem Compound (Dec 2012). The maximum degree required to perceive the ring systems was measured for each system. An arbitrarily high value of (*deg* = 5000) was chosen as an absolute maximum value. It was found that 987 (0.005%) systems would require a threshold larger than maximum 5000 to finish. Retrospectively calculating the threshold required for a given percentile showed only a small gain for higher values (Table 9). Even using a small threshold of only 9 allows perception of 99% of the ring systems (Figure 13). The default value (used in benchmarks) was chosen as 684 which from this test would allow the algorithm to feasibly complete on ∼99.99*%* of the systems present in PubChem Compound (Dec 2012).

**Table 9:** Ring systems (PubChem-Compound) that were feasibly handled by the improved AllRingsFinder at different thresholds

**Percentile**	**Threshold (*degree*)**	**Feasible**	**Infeasible**
		**ring systems**	**ring systems**
99.95	72	17,834,013	8,835
99.96	84	17,835,876	6,972
99.97	126	17,837,692	5,156
99.98	216	17,839,293	3,555
99.99	684 (default)	17,841,065	1,783
99.991	882	17,841,342	1,506
99.992	1,062	17,841,429	1,419
99.993	1,440	17,841,602	1,246
99.994	3,072	17,841,789	1,059
99.9946	5,000 (max tested)	17,841,861	987

**Figure 14:**
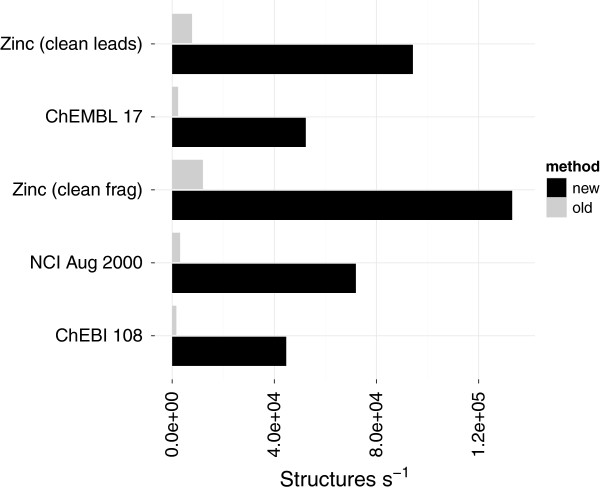
**Performance comparison when finding all elementary cycles.** The performance difference between the old and new implementations for finding all elementary cycles. The performance includes conversion to an *incidence* list representation (Table 2).

The new threshold still encounters infeasible structures but number found is fewer and does not vary between runs (Table 10). The performance of the new implementation was compared against the old algorithm using a time out of 5 ms (much lower than default). With the small timeout 2,500 structures in chembl_17 were considered infeasible by the old implementation whilst using the new implementation with the default threshold only 232 are infeasible. The new algorithm was able to compute more cycles (Table 11) in less time (Figure 14). The time taken to find all cycles in chembl_17 previously took ∼9 minutes (with a small threshold) but now takes only ∼25 seconds (Table 12). Disregarding the conversion we found that when the computation was feasible, determining all cycles was as fast as the smaller subsets.

**Table 10:** **Average (*n* = 15) number of structures considered infeasible by the old and new implementations**

**Chemical**	***n* structures**	***n* fail (old)**	***n* fail (new)**		
**structure**					
chebi_108	26,790	108-117	41		
nci_aug00	250,172	306-311	37		
zinc_frag	504,074	0	0		
chembl_17	1,318,180	2528-2547	232		
zinc_leads	5,135,179	0	0		

Based on a timeout procedure the number of cycles found in the old procedure varied between repeats, given the same structures the new feasibility threshold does not.

**Table 11:** **Average (*n* = 15) number of all cycles found in each datasets**

**Chemical structure**	***n* structures**	**Old**		**New**	
		**Cycles**	**sdev**	**Cycles**	**sdev**
chebi_108	26,790	98,597	199	126,713	0
nci_aug00	250,172	936,625	409	1,007,643	0
zinc_frag	504,074	1,022,498	0	1,022,498	0
chembl_17	1,318,180	6,176,585	378	6,599,942	0
zinc_leads	5,135,179	14,816,752	0	14,816,752	0

Due to the time out the old implementation sometimes provided different counts for each repeat.

**Table 12:** **Average and median (*n* = 15) time taken to find all rings using existing and improved implementations of AllRingsFinder**

**Chemical structure**	**Old**			**New**		
	***t* (ms)**	**median**	**sdev**	***t* (ms)**	**median**	**sdev**
		***t* (ms)**			***t* (ms)**	
chebi_108	16,293	16,179	540	599	574	105
nci_aug00	80,417	80,339	319	3,478	3,449	143
zinc_frag	41,854	41,747	458	3,786	3,778	60
chembl_17	568,984	568,809	829	25,200	25,181	99
zinc_leads	661,028	661,368	1133	54,490	54,471	101

The newer code includes the overhead of conversion to (and from) the CDK objects. (Table 2). Unprocessed measurements are provided in Additional file 3: All cycles.

### Additional cycles sets

The shortest cycle through each vertex and edge is also provided as a unique but potentially exponential cycle set. The edge-short cycles has also been termed the Largest Set of Smallest Rings (LSSR) and is utilised within Open Babel [28]. Computation of the sets does not check if the cycles form a basis. This could improve performance but no noticeable change was observed in measurements. The implementations are provided for compatibility.

A TripletCycles utility was also implemented to improve generation of CACTVS [29] Substructure Keys (PubChemFingerprint). These cycles are the shortest through a vertex triple {*u*,*v*,*w*} and allows generation of cycles for envelope rings such as naphthalene or azulene whilst avoiding larger fused rings. The implementation allows a unique or non-unique set to be generated.

## Conclusion

The improved performance in cycle perception means it is now feasible to analyse much larger chemical data sets. This is particularly true of the unique short cycle sets (essential and relevant) which saw an order of magnitude improvement. It is now no longer favourable to utilise the non-unique MCB due to runtime performance. Any procedures incorrectly relying on the MCB to be unique can be easily adapted to use the new algorithms. The efficient implementation of the relevant cycles could also be adapted to compute a recent descriptor known as Unique Ring Families [30].

Improvements were seen throughout the toolkit with cycle perception being required for core functionality. The new algorithm for cycle membership has been used to improve performance of atom typing and the set of all cycles utilised in aromaticity perception. To avoid a performance hit from the old implementation the aromaticity of non-shortest cycles was only perceived for small fused rings systems. The new aromaticity has no restrictions and attempts to perceive aromaticity on all cycles. If computation is not feasible the aromaticity perception falls back to a smaller more feasible cycle set. Alternatively the smaller set of cycles could be tested first with the larger set only utilised if potentially aromatic atoms were remaining. Using the optimised representations the set of all cycles is generally faster to compute than the smaller sets and it is preferable to try all and fail fast.

A large portion of the time is spent in converting the CDK objects to optimised representations. Despite this without the conversion the runtime performance is much slower. Further gains could be made by optimising the native data structures and removing the need for conversion. The changes required would be large but could be introduced in future releases.

## Availability and requirements

**Project Name:** The Chemistry Development Kit**Project Home Page:**http://sourceforge.net/projects/cdk/ (version CDK (development)) or http://github.com/cdk/cdk (version 1.5.4 onwards)**Operating System:** Platform Independent**Programming Language:** Java**Requirements:** Java 1.6+**License:** Lesser General Public License 2.1

## Endnotes

^a^ alternatively known as cyclomatic number, nullity (*μ*), frère jacque number, first Betti’s number or bond closures [5].

^b^ linear independence is check with row reduction of a matrix (Gaussian elimination).

## Abbreviations

The following abbreviations have been used in reference to implementation class names in the CDK library. Here we detail the full package name used in library. **AllRingsFinder:**http://org.openscience.cdk.ringsearch.AllRingsFinder; **Cycles:**http://org.openscience.cdk.graph.Cycles; **MinimumCycleBasis:**http://org.openscience.cdk.graph.MinimumCycleBasis; **PubChemFingerprint:**http://org.openscience.cdk.fingerprint.PubChemFingerprint; **RingSearch:**http://org.openscience.cdk.ringsearch.RingSearch; **SSSRFinder:**http://org.openscience.cdk.ringsearch.SSSRFinder; **SpanningTree:**http://org.openscience.cdk.graph.SpanningTree; **TripletCycles:**http://org.openscience.cdk.graph.TripletCycles.

## Competing interests

The authors declare that they have no competing interests.

## Authors’ contributions

JM implemented, benchmarked the ring perception algorithms and wrote the manuscript. CS designed the core library and provides continuous support. Both authors read and approved the final manuscript.

## Supplementary Material

Additional files 1 **Cycle membership benchmark.** Performance measurements of determining cycle membership using SpanningTree and RingSearch. Click here for file

Additional files 2 **Short cycles.** Performance measurements of determining short cycles (MCB, essential and relevant) using the old and new implementations. Click here for file

Additional files 3 **All cycles.** Performance measurements of determining all cycles using the old and new implementations. Click here for file
